# SLC25A10 promotes cisplatin resistance by inhibiting ferroptosis in cervical cancer

**DOI:** 10.1038/s41420-025-02712-5

**Published:** 2025-10-07

**Authors:** Chenglei Ma, Xiaoyi Lu, Chen Ni, Yu Gao, Fei Yang, Shiwen Chen, Yi Du, Fang Zhao, Ying Cao, Haiwei Huang

**Affiliations:** 1 Obstetrics and Gynecology Department, The Affiliated Zhangjiagang Hospital of Soochow University, Suzhou, China; 2 Oncology Department, The Affiliated Zhangjiagang Hospital of Soochow University, Suzhou, China; 3 The Affiliated Zhangjiagang Hospital of Soochow University, Suzhou, China

**Keywords:** Cancer metabolism, Mechanisms of disease, Cervical cancer

## Abstract

Cisplatin (DDP)-based chemotherapy is the standard first-line treatment for cervical cancer (CC). However, many patients with CC develop resistance to DDP, either initially or over time. This resistance significantly limits the effectiveness of treatment. Therefore, identifying new therapeutic targets and combination therapies to overcome DDP resistance is a critical need. In this study, we investigated the expression of SLC25A10 in cervical cancer tissues using bioinformatics analysis and partial tissue analysis. We found that SLC25A10 expression was significantly higher in human cervical cancer tissues compared to normal tissues, based on data from The Cancer Genome Atlas (TCGA) and clinical samples. Moreover, increased SLC25A10 expression was associated with adverse clinicopathological characteristics of cervical cancer patients. To explore the functional role of SLC25A10, we conducted a series of in vitro and in vivo experiments. Our results demonstrated that SLC25A10 promotes cervical cancer cell growth, migration, and resistance to DDP. Mechanistically, we found that inhibiting SLC25A10 expression restricted the transport of glutathione (GSH) and reduced the expression of glutathione peroxidase 4 (GPX4). This led to increased intracellular lipid peroxidation and accumulation of reactive oxygen species (ROS), ultimately promoting iron-mediated cell death (ferroptosis) in cervical cancer cells. In conclusion, our findings suggest that SLC25A10 may serve as a novel therapeutic target to overcome cisplatin resistance and enhance the efficacy of chemotherapy in CC. Future studies should focus on further elucidating the role of SLC25A10 in CC and exploring its potential as a therapeutic target in combination with other treatments.

## Introduction

Cervical cancer is a serious threat to women’s health worldwide [1, 2]. Each year, approximately 570,000 women are diagnosed with cervical cancer worldwide, and more than 311,000 women die from this disease; moreover, developing countries account for more than 85% of all cases. Platinum-based chemotherapy is the standard treatment for cervical cancer. Cisplatin disrupts the template function of the DNA double helix by forming intrachain and interchain adducts that result in DNA damage [3]. Through this mechanism, cisplatin inhibits DNA replication and transcription, which leads to the apoptosis of cancer cells [4]. However, the remission rate of patients with recurrent and advanced cervical cancer who are treated with chemotherapy is only approximately 25%, and the average survival time of patients is less than 1 year [5]. One confounding factor that affects treatment success is resistance to platinum-based drugs [6]. Possible mechanisms of resistance to platinum-based drugs have been proposed, including increased DNA repair, decreased platinum uptake, increased platinum efflux, increased platinum inactivation, and inhibition of apoptosis [7–11].

The mitochondrial carrier family (SLC25) consists of 53 members and is the largest solute transport protein family in humans. These proteins transport solutes across the impermeable inner membrane of mitochondria, facilitating important cellular processes such as the oxidative phosphorylation of fats and sugars, amino acid catabolism and interconversion, synthesis of iron-sulfur clusters and heme, macromolecular synthesis, and thermogenesis. Abnormal expression of SLC25 family proteins can lead to metabolic disturbances, thereby contributing to the development of both cancerous and non-cancerous diseases [12]. Therefore, a systematic study of the role of SLC25 family proteins in CC will help identify more potential therapeutic targets and prognostic markers for the disease. Among them, SLC25A10 encodes proteins that exchange dicarboxylate (e.g., malate, succinate) into phosphates, sulfates, and other small molecules in mitochondrial membranes, thus providing substrates for metabolic processes such as the Krebs cycle and fatty acid synthesis [13]. Evidence suggests that SLC25A10 is involved in both energy metabolism and redox homeostasis. Increased SLC25A10 expression has been demonstrated in a variety of tumors, but the role of SLC25A10 in cervical cancer has not been reported.

Ferroptosis is an iron-dependent form of cell death that is mechanistically distinct from apoptosis, necroptosis, and other types of regulated cell death. After its discovery in 2012 by Dixon et al., ferroptosis quickly gained attention in the context of cardiovascular and neurodegenerative diseases and cancer [14]. Studies have shown that certain conditions predispose cells to ferroptosis. For example, mesenchymal cells are more sensitive to ferroptosis induction than epithelial cells because of the increased content of easily oxidized polyunsaturated fatty acids in the cell membranes of mesenchymal cells [15]. Thus, ferroptosis may be a potential strategy by which metastatic cancer cells can be eliminated. In addition, immunotherapy-resistant subtypes of dedifferentiated melanoma cells are susceptible to ferroptosis due to reduced glutathione levels [16]. Reduced levels of glutathione and NADPH have also been observed in drug-resistant tumor cell populations, which suggests that these compounds are targets of ferroptosis inducers [17]. Finally, unstable iron pools in the cytoplasm of cancer stem cells are increased, and consequently, ferroptosis is highly likely to occur [18]. Therefore, ferroptosis can eliminate the subpopulation of tumor cells responsible for cancer recurrence. To improve their anticancer effects, ferroptosis can be combined with chemotherapy [19], radiation therapy [20], photodynamic therapy [21], and immunogenic cell death with immunotherapy [22].

In this study, we found that SLC25A10 can induce intracellular lipid peroxidation and the accumulation of reactive oxygen species by restricting GSH transport, thus promoting ferroptosis in cervical cancer cells. This suggests that SLC25A10 may serve as a molecular marker of cisplatin resistance in cervical cancer and as a new target for improving cisplatin efficacy.

## Results

### SLC25A10 is upregulated in cervical cancer

**Fig. 1:**
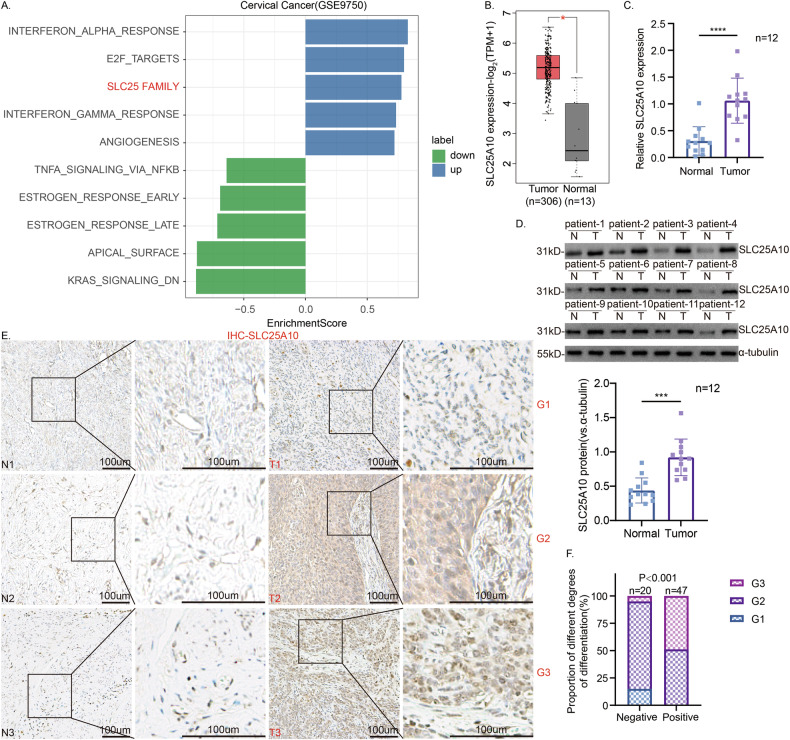
SLC25A10 is highly expressed in the tissues of patients with cervical cancer. We conducted Gene Set Enrichment Analysis (GSEA) using the Hallmarks gene set and identified numerous pathways significantly associated with cervical cancer (*p*-value ≤ 0.05). Among these, the SLC25 family gene set was significantly enriched (*P* = 0.02995) and ranked third among the upregulated Hallmark pathways (**A**). TCGA database analysis revealed that SLC25A10 expression was upregulated in cervical cancer (http://gepia.cancer-pku.cn) (**B**). mRNA and protein measurements were performed on cervical cancer tissue (“T”) and matched normal cervical epithelial tissue (“N”) from 12 (*n* = 12) patients with primary cervical cancer, and the results were quantified (**C**, **D**). Three representative SLC25A10-stained images of cervical cancer tissues and matched paracancerous cervical epithelial tissues, including highly differentiated G1, moderately differentiated G2, and poorly differentiated G3 tissues are shown (**E**). The correlation between SLC25A10 expression and the degree of tumor differentiation was statistically analyzed (**F**). * Compared with “T”, *P* < 0.05. Scale =100 µm.

Initially, we downloaded and analyzed the GSE9750 dataset using the limma package for differential expression, followed by Gene Set Enrichment Analysis (GSEA) with the Hallmarks gene set, and identified numerous pathways significantly associated with cervical cancer (*p*-value ≤ 0.05). The SLC25 family gene set was significantly enriched (*P* = 0.02995) and ranked third among the upregulated Hallmark pathways (Figs. 1A and S1A). Subsequently, we analyzed the differential expression patterns of 53 SLC25 family proteins in CC using the TCGA database. By comparing cervical cancer patients with the normal population, we identified 12 SLC25 family proteins with statistically significant differences. Among them, 5 genes (SLC25A5, SLC25A8, SLC25A10, SLC25A39, and SLC25A50) exhibited significant upregulation in cervical cancer tissues, while the other 7 genes showed downregulation (Figs. 1B and S1B). Considering the targeting and convenience for subsequent drug development, we focused further on these 5 upregulated genes. To verify their expression characteristics in CC, we detecteded their mRNA levels in human cervical cancer tissues. The results indicated that SLC25A10 had the most significant difference in mRNA expression between cervical cancer tissues and the control group (*n* = 12, Figs. 1C and S2). Therefore, SLC25A10 may be involved in the development of CC. Combined with the Western blotting data, we also found that the protein expression of SLC25A10 was significantly upregulated in the tissues from these 12 cervical cancer patients (Fig. 1D). In addition, we performed IHC on tissues from 67 patients, including the above 12 patients, and obtained SLC25A10 staining images of 3 pairs of representative cervical cancer tissues and matched paracancerous cervical epithelial tissues, including highly differentiated G1, moderately differentiated G2, and poorly differentiated G3 tissues (Fig. 1E). The results revealed that SLC25A10 expression was significantly correlated with the degree of tumor differentiation and that poorly differentiated cervical cancer tissues were significantly more likely to be SLC25A10-positive (Fig. 1F). The above 67 patients with CC were further divided into SLC25A10-positive and SLC25A10-negative groups according to the IHC results, and the relationships between SLC25A10 expression and the clinicopathological characteristics of the patients were analyzed. The results revealed that the expression level of SLC25A10 was closely related to the degree of tumor differentiation, the degree of lymph node metastasis and the FIGO stage (*P* < 0.05). However, the SLC23A10 expression level was not related to age or histopathologic type (*P* > 0.05) (Table 1). These data suggest that SLC25A10 may be involved in the malignant development of CC.

**Table 1:** Relationship between SLC25A10 and clinicopathological features in patients with cervical cancer (case (%)).

Parameters		*n*	SLC25A10		χ^2^, *p*
			Negative (*n* = 20)	Positive (*n* = 47)	
Age	≤58 years old	39	14 (35.9)	25(64.1)	1.629, 0.202
	>58 years old	28	6 (21.4)	22 (78.6)	
Histopathological type	Squamous	48	14 (29.2)	34 (70.8)	0.038, 0.846
	Nonsquamous	19	6 (31.6)	13 (68.4)	
Differentiation	High	3	3 (15.0)	0 (0.0)	16.803, <0.001***
	Middle	40	16 (80.0)	24 (51.1)	
	Low	24	1 (5.0)	23 (48.9)	
Lymph node metastasis	No	48	19 (39.6)	29 (60.4)	6.105, 0.013*
	Yes	19	1 (5.3)	18 (94.7)	
FIGO Stage	I	32	18 (56.3)	14 (43.8)	20.297, <0.001***
	II	28	2 (7.1)	26 (92.9)	
	III-IV	7	0 (0.0)	7 (100.0)	

Clinicopathological features were assessed using the Fisher’s exact test. *P < 0.05, ***P < 0.001.

### Overexpression of SLC25A10 promotes the growth and migration of cervical cancer cells

**Fig. 2:**
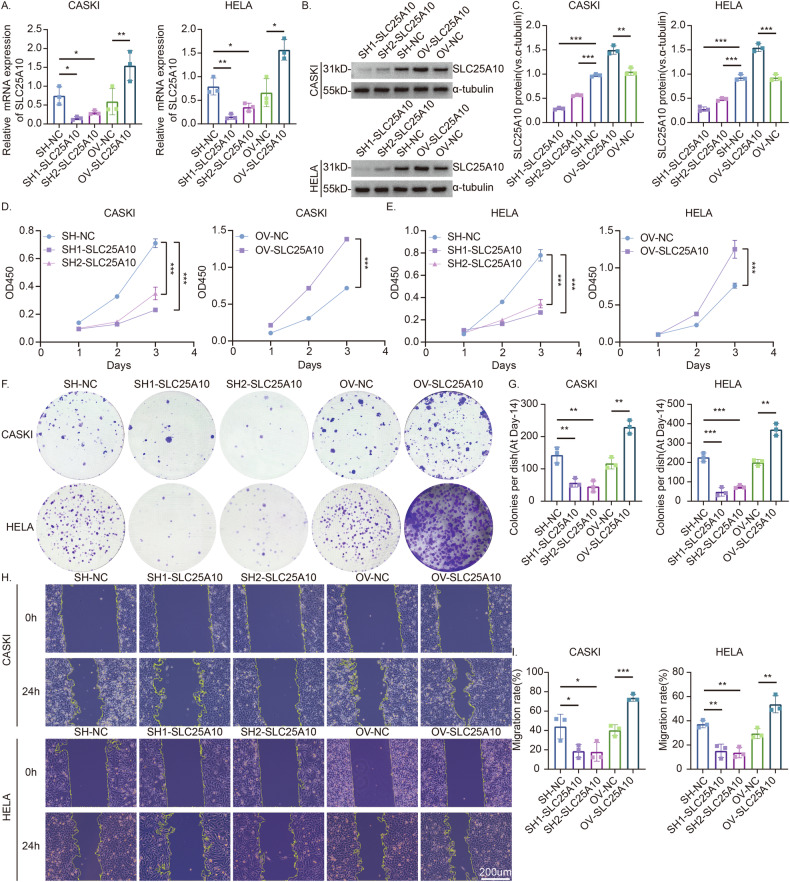
Overexpression of SLC25A10 can promote the proliferation and migration of cervical cancer cells. Lentiviruses were transfected into CaSki and HeLa cells, and a knockdown group (“SH1/SH2”, which represent two different sequences) and its control SH-NC were constructed; similarly, an overexpression group (OV) and its control OV-NC were also constructed. The mRNA and protein levels were detected, and protein levels were semiquantified (**A**–**C**). Cell viability was determined by CCK-8 assay. After SLC25A10 knockdown, cell viability was decreased significantly but was increased in the SLC25A10-overexpression group (**D**, **E**). A colony formation assay was used to assess cell proliferation. After SLC25A10 silencing, cell proliferation slowed, whereas cells in the SLC25A10-overexpression group formed more colonies (**F**, **G**). A quantitative measurement using a scratch test to detect cell migration revealed that SLC25A10 silencing slowed cell migration in vitro, whereas SLC25A10 upregulation increased cell migration compared with that in the control group (**H**, **I**). Compared with the control group, *P < 0.05, **P < 0.01, ***P < 0.001, ****P < 0.0001. Scale =200 µm. The data represent the means ± SDs.

To test whether SLC25A10 exerts cancer-promoting effects, we first constructed cervical cancer cell lines that stably overexpress SLC25A10 and then knocked down SLC25A10 in CaSki and HeLa cervical cancer cells using a lentivirus plasmid or a specific shRNA. Validation revealed that SLC25A10 mRNA was significantly decreased in CaSki and HeLa cells in the knockdown group but significantly increased in cells in the overexpression group (Fig. 2A). The protein levels were also consistent with this result (Fig. 2B, C). Furthermore, the CCK-8 experiment showed that after SLC25A10 was knocked down, cell viability decreased, while in the SLC25A10-overexpressing group, cell viability was significantly increased (Fig. 2D, E). A colony formation assay also confirmed that the proliferation of SLC25A10-silenced cells was decreased compared with that of control cells, while the proliferation of SLC25A10-overexpressing cells was significantly accelerated (Fig. 2F, G). According to scratch tests, we also found that targeted silencing of SLC25A10 slowed the migration of CaSki and HeLa cells in vitro, whereas the cell migration rate was significantly greater after upregulation of SLC25A10 than that in the control group (Fig. 2H, I). These results suggest that SLC25A10 plays a carcinogenic role in CC.

### SLC25A10 enhances cervical cancer resistance to cisplatin

**Fig. 3:**
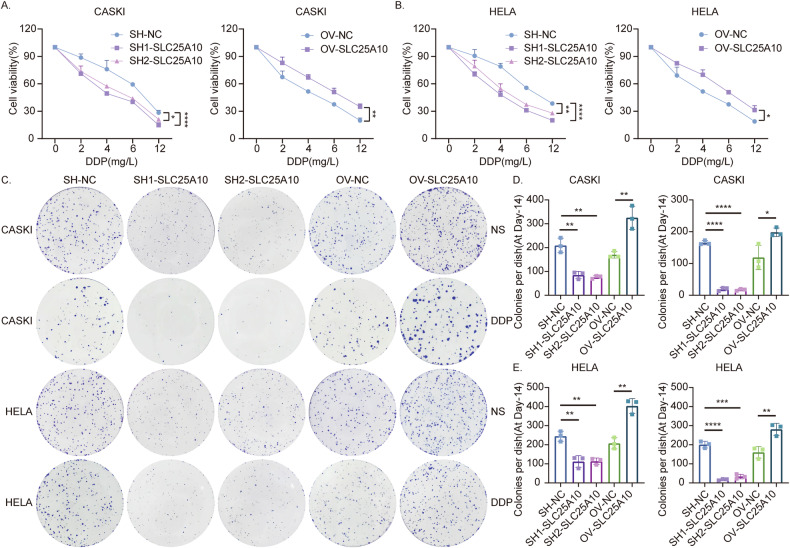
Overexpression of SLC25A10 can increase the resistance of cervical cancer cells to cisplatin. After 24 h of treatment with different concentrations of DDP, the viability of SLC25A10-overexpressing cells and those with SLC25A10 knockdown was determined by CCK-8 assay (**A**, **B**). Cervical cancer cells treated with 4 mg/DDP for 16 h and cultured for 2 weeks were cloned, and the results were quantified (**C**–**E**). Compared with the control group, *P < 0.05, **P < 0.01, ***P < 0.001, and ****P < 0.0001. Scale =200 µm. The data represent the means ± SDs.

The effectiveness of chemotherapy for advanced CC ranges from 20% to 36%, and the overall survival/prognosis of patients with recurrence and metastasis is very poor [5]. Therefore, we further investigated whether changes in SLC25A10 expression affect the sensitivity of cervical cancer cells to cisplatin. First, the constructed CaSki and HeLa cell lines in which SLC25A10 was either knocked down or overexpressed were exposed to different concentrations of DDP, after which CCK-8 and colony formation assays were used to evaluate the viability and proliferation ability of the cells, respectively (Fig. 3A–E). The CCK-8 assay revealed a corresponding increase in cell mortality in SLC25A10-knockdown cells compared with control CaSki and HeLa cells treated with DDP; in contrast, SLC25A10-overexpressing CaSki and HeLa cells exhibited better survival under DDP stimulation than did control cells (Fig. 3A, B). A colony formation assay also confirmed that compared with control cells treated with DDP, SLC25A10-overexpressing cells were less sensitive to DDP and formed more colonies after 2 weeks of culture. In contrast, under DDP stimulation, CaSki and HeLa cells with SLC25A10 knockdown exhibited worse survival than control cells and formed significantly fewer colonies than DDP-treated control cells (Fig. 3C–E). These results suggest that increased SLC25A10 expression inhibits DDP-induced cell death and endows cervical cancer cells with resistance to DDP.

### SLC25A10 promotes cervical cancer by inhibiting ferroptosis

**Fig. 4:**
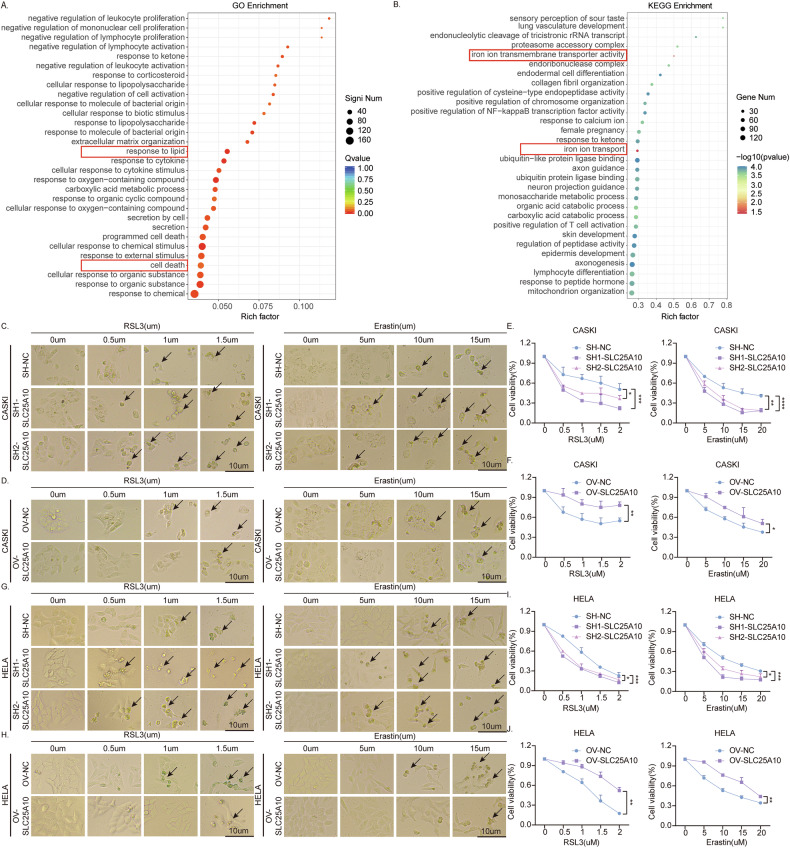
SLC25A10 inhibits ferroptosis in cervical cancer cells. GO analysis revealed that SLC25A10 is involved in various types of cellular metabolism, including lipid metabolism, oxidation‒reduction, carboxylic acid metabolism, and programmed cell death (**A**). KEGG analysis suggested that SLC25A10 is associated with iron ion transmembrane transporter activity and iron ion transport (**B**). The death of CaSki and HeLa cells with SLC25A10 overexpression or knockdown was detected after 24 h of treatment with different concentrations of RSL3 or erastin (**C**–**J**). The morphological changes in the cells were observed by microscopy (**C**, **D**, **G**, **H**). The viability of cells with either high or low SLC25A10 expression was evaluated by CCK-8 assay (**E**, **F**, **I**, **J**). Compared with the control group, *P < 0.05, **P < 0.01, ***P < 0.001, and ****P < 0.0001. Scale =10 µm. The data represent the means ± SDs.

To explore the potential mechanism of the tumorigenic action of SLC25A10 in cervical cancer cells, we first performed transcriptome sequencing (RNA-seq) of SH1-SLC25A10 and SH-NC HeLa cells. GO analysis revealed that SLC25A10 is involved in metabolism, including lipid metabolism, oxidation‒reduction, carboxylic acid metabolism, and programmed cell death, in a variety of cell types (Fig. 4A). KEGG analysis also suggests that SLC25A10 is associated with iron ion transmembrane transporter activity and iron ion transport (Fig. 4B). Previous studies have confirmed that cisplatin could induce ferroptosis [23–25]. Based on the GO and KEGG enrichment results and the role of cisplatin in ferroptosis, SLC25A10 appears to be closely related to ferroptosis. Further, to explore whether SLC25A10 plays a role in the regulation of ferroptosis, we first exposed the constructed SLC25A10-knockdown and SLC25A10-overexpressing CaSki cells to different concentrations of ferroptosis inducers (RSL3 or erastin) for 24 h. The inhibitory effects of RSL3 or erastin on cell viability were evaluated by cell morphology analysis and a CCK-8 assay (Fig. 4C–F). Compared with that in control CaSki cells, cell mortality in SLC25A10-knockdown cells was increased after exposure to different concentrations of RSL3 or erastin; in contrast, CaSki cells overexpressing SLC25A10 exhibited better survival under RSL3 or erastin stimulation than control cells. Similar experimental results were also obtained in HeLa cells (Fig. 4G–J). Therefore, we speculate that SLC25A10 may exert its cancer-promoting effects by inhibiting ferroptosis.

### SLC25A10 increases cervical cancer resistance to cisplatin by inhibiting ferroptosis

**Fig. 5:**
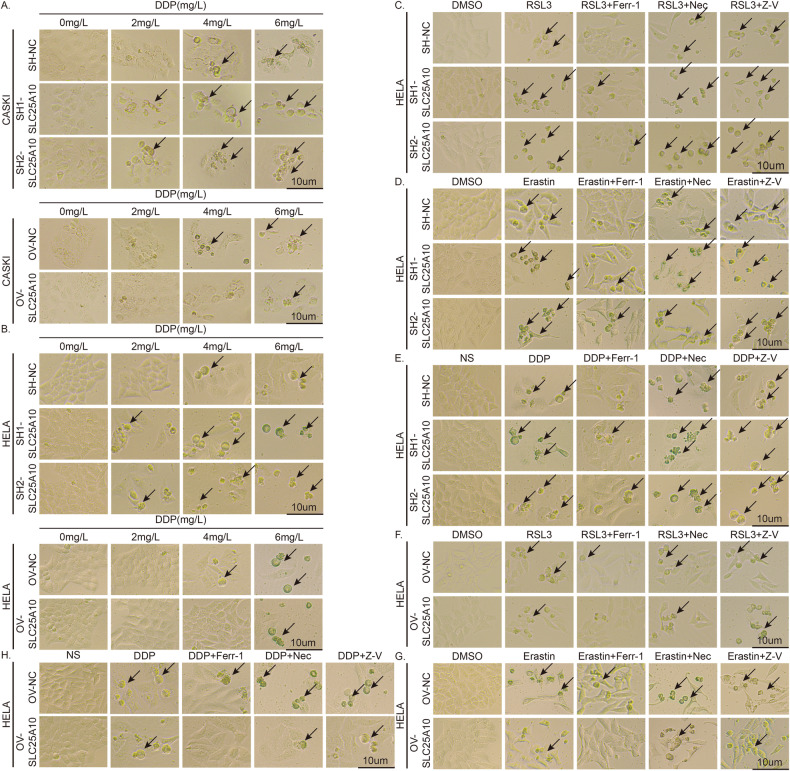
SLC25A10 promotes DDP resistance in cervical cancer cells by inhibiting ferroptosis. Cell viability was assessed via light microscopic analysis (**A**–**H**). After 24 h of treatment with different concentrations of DDP, less cell death of CaSki and HeLa cells overexpressing SLC25A10 was observed, whereas more cell death was observed in the knockdown group (**A**, **B**). HeLa cells in which SLC25A10 was overexpressed or knocked down were treated with 1 µM RSL3, 10 µM erastin or 4 µg/mL DDP for 24 h, and DMSO or NS was used as a control. We found that apoptosis (Z-V) and necrosis (Nec) inhibitors could not prevent RSL3- and erastin-induced cell death. However, a ferroptosis inhibitor (Ferr-1) partially attenuated ferroptosis inducer-induced cell death (**C**, **D**, **F**, **G**). After the ferroptosis inducer was substituted with DDP, cell death was also reversed by the ferroptosis inhibitor, independent of the use of the apoptosis and necrosis inhibitors (**E**, **H**). Scale =10 µm.

Previous studies have confirmed that DDP can induce ferroptosis in cells [23–25]. Does SLC25A10 affect cervical cancer cell tolerance to DDP by mediating ferroptosis? To answer this question, we exposed the constructed SLC25A10-knockdown or SLC25A10-overexpressing CaSki and HeLa cells to different concentrations of DDP for 24 h (Fig. 5A, B) and found that the observed cell death was consistent with the previous CCK-8 results (Fig. 3A, B). Considering the good response of HeLa cells to drugs, we selected HeLa cells for the next experiment. We treated SLC25A10-overexpressing and SLC25A10-knockdown HeLa cells with 1 µM RSL3, 10 µM erastin or 4 μg/mL DDP for 24 h to investigate the effects of different inhibitors on induced cell death (Fig. 5C–H). We found that apoptosis (Z-V) and necrosis (Nec) inhibitors could not prevent RSL3- and erastin-induced cell death. However, a ferroptosis inhibitor (Ferr-1) partially attenuated iron-induced cell death (Fig. 5C, D, F, G). After the substitution of ferroptosis inducers with DDP, cell death could also be reversed by ferroptosis inhibitors without the effects of apoptosis and necrosis inhibitors (Fig. 5E, H). These results suggest that SLC25A10 promotes DDP resistance in cervical cancer cells by inhibiting ferroptosis.

### SLC25A10 regulates ferroptosis by inhibiting GSH transport and GPX4 expression

**Fig. 6:**
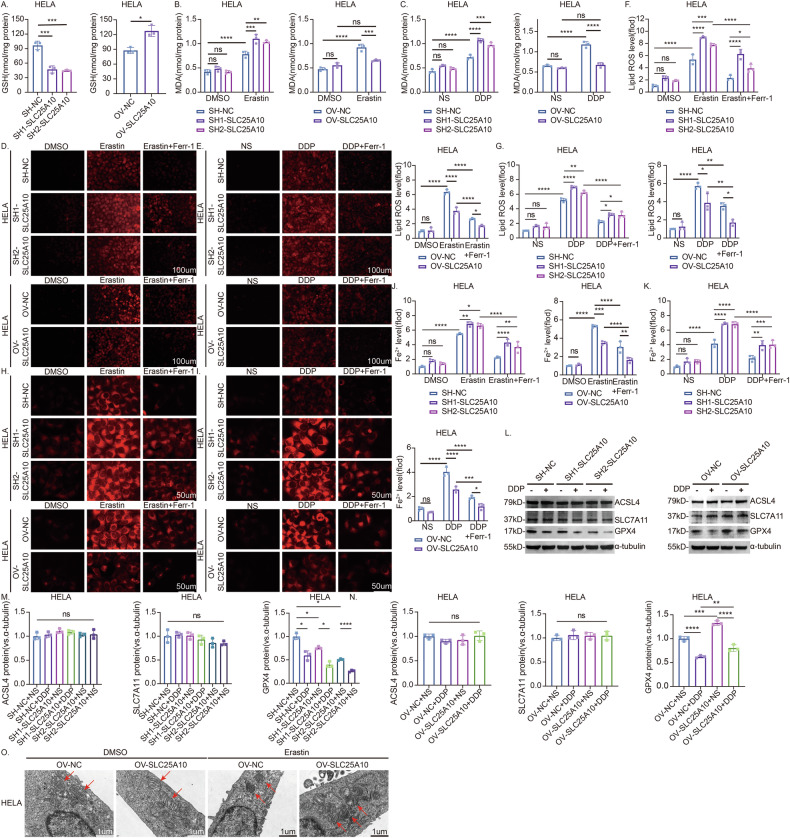
SLC25A10 affects the resistance of cervical cancer cells to ferroptosis by reducing GSH and GPX4. Quantitative studies of the GSH content in HeLa cells revealed that the GSH content decreased after SLC25A10 knockdown, whereas the GSH content increased in SLC25A10-overexpressing cells (**A**). Quantitative studies on the MDA content in HeLa cells treated with erastin or DDP revealed that the MDA content increased more in cells with low SLC25A10 expression than in control cells; in contrast, the MDA content did not increase significantly in the group with high SLC25A10 expression, and the difference was not statistically significant. DMSO or NS was used as a control (**B**, **C**). Quantitative analyses of ROS levels in HeLa cells treated with erastin or DDP, with or without Ferr-1. Under erastin or DDP stimulation, the ROS levels in the knockdown group were significantly greater than those in the control group, whereas those in the overexpression group were relatively lower. This phenomenon was reversed by Ferr-1. DMSO or NS was used as a control (**D**–**G**). Quantitative studies on the changes in Fe^2+^ levels in HeLa cell lines treated with erastin or DDP, with or without Ferr-1. Under stimulation with erastin or DDP, the knockdown group exhibited significantly higher Fe^2+^ levels than the control group, while the overexpression group had relatively lower levels. This phenomenon was reversed by Ferr-1. DMSO or NS was used as a control (**H**–**K**). Additionally, we investigated the effect of cisplatin on the expression of GPX4, ACSL4, and SLC7A11 in HeLa cell lines by detecting protein expression levels and quantifying the results (**L**–**N**). HeLa control cells or SLC25A10-overexpressing cells were treated with 10 µM erastin for 24 h and then prepared for transmission electron microscopy. The control group exhibited typical mitochondrial changes associated with ferroptosis, that is, a reduced or absent mitochondrial ridge, increased membrane density, and increased permeability. The morphological changes in the mitochondria of the SLC25A10-overexpressing cells were less substantial (**O**). Compared with the control group, ns: not significant, *P < 0.05, **P < 0.01, ***P < 0.001, ****P < 0.0001, *n* = 3. Scale =100 µm, 50 µm and 1 µm. The data represent the means ± SDs.

The occurrence of ferroptosis is primarily due to intracellular oxidative damage and an imbalance in reductive protection. The classical ferroptosis inhibition pathway occurs through the cystine/glutamate antiporter transporter (systemXC) and glutathione peroxidase 4 (GPX4), which promote glutathione (GSH) production and lipid peroxidase consumption. GSH is an intracellular antioxidant that can protect cells from reactive oxygen species (ROS), and high levels of GSH can promote cancer cell survival and resistance to chemotherapy [19]. It has been confirmed that SLC25A10 is a transporter of GSH [26, 27]. Our study also confirmed that GSH expression was decreased in HeLa cells after SLC25A10 knockdown, whereas GSH was increased in SLC25A10-overexpressing cells (Fig. 6A). MDA is a product of ferroptosis, and its expression increases with increasing ferroptosis [28]. After erastin treatment, MDA expression was increased in all the cells. The level of MDA in HeLa cells with SLC25A10 knockdown was significantly greater than that in control cells. In contrast, the MDA content in the high-expression group was significantly lower than that in the control group (Fig. 6B). Moreover, the changes in the MDA content in response to DDP also showed the same trend in HeLa cells (Fig. 6C). We also detected ROS levels in the cells. Under fluorescence microscopy, the ROS-positive cells were red, and their brightness was proportional to the level of ROS. Under erastin or DDP stimulation, the brightness of the DCFH-DA probe in the knockdown group was significantly greater than that in the control group, whereas the brightness in the overexpression group was lower than that in the control group. This phenomenon was reversed by Ferr-1 (Fig. 6D–G).

Under the same stimulation of erastin or DDP, silencing SLC25A10 significantly increases intracellular Fe^2+^ accumulation, while overexpression of SLC25A10 significantly reduces intracellular Fe^2+^ levels. This effect can also be reversed by Ferr-1. These results suggest that SLC25A10 may influence intracellular Fe^2+^ content during erastin- or DDP-induced ferroptosis in cervical cancer cells (Fig. 6H–K). At the same time, we speculate that SLC25A10’s regulation of ferroptosis may also involve other mechanisms, such as regulating the expression or activity of key proteins in the ferroptosis pathway. To verify this hypothesis, we detected the expression of several key proteins in the ferroptosis pathway in cells. The mRNA expression levels and protein immunoblotting showed that under the action of DDP, the expression of GPX4 in cervical cancer cells decreased, indicating that DDP may induce ferroptosis by affecting GPX4 levels. After silencing SLC25A10, GPX4 expression was significantly reduced. However, overexpression of SLC25A10 partially restored GPX4 protein levels, indicating that downregulating SLC25A10 can further reduce GPX4 expression and exacerbate DDP-induced ferroptosis in cervical cancer cells. On the contrary, overexpression of SLC25A10 can alleviate DDP-induced ferroptosis by restoring GPX4 expression. However, the expression levels of ACSL4 and SLC7A11 were not significantly affected during this process (Figs. 6L–N and S3). Decreased mitochondrial volume, a reduction in or disappearance of mitochondrial cristae, and rupture of the plasma and mitochondrial membranes are typical morphological characteristics of ferroptosis, which suggests mitochondrial dysfunction [29]. Furthermore, erastin was used to treat HeLa cells overexpressing SLC25A10 and corresponding control cells. Using electron microscopy, we observed that the control group exhibited typical mitochondrial changes associated with ferroptosis, such as fewer and smaller mitochondrial cristae and increased mitochondrial permeability. The morphological changes in the mitochondria of the SLC25A10-overexpressing cells were less substantial (Fig. 6O). In conclusion, our data suggest that knockdown of SLC25A10 knockdown reduces intracellular GSH levels and GPX4 content, thereby weakening the resistance of cervical cancer cells to cisplatin-induced ferroptosis.

### Depletion of SLC25A10 enhances cervical cancer sensitivity to cisplatin in vivo

**Fig. 7:**
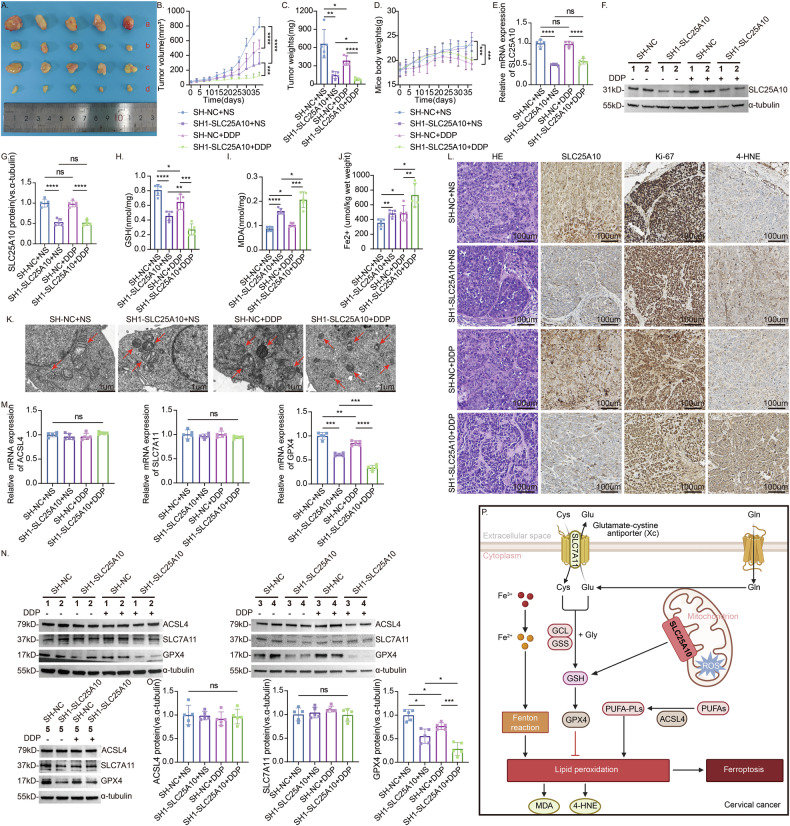
Knockdown of SLC25A10 enhances the sensitivity of subcutaneous tumors to cisplatin in nude mice. Xenograft tumors in the four groups of nude mice after subcutaneous injection of SLC25A10-knockdown HeLa cells or their control cells and treatment with DDP (3 mg/kg) or NS (**A**): (a) SH-NC + NS group (*n* = 5), (b) SH-NC + DDP group (*n* = 5), (**c**) SH1-SLC25A10 + NS group (*n* = 5), and (d) SH1-SLC25A10 + DDP group (*n* = 5). Body weight of mice, and the volume and weight of the subcutaneous xenograft tumors (**B**–**D**). The in vivo knockout effect was evaluated by detecting SLC25A10 mRNA and protein levels in tumor tissue, and the results were quantified (**E**–**G**). A quantitative study of the GSH content in xenograft tumor tissues revealed that the GSH content in SLC25A10-knockdown tumors was lower than that in the control group and that the GSH content was further reduced after DDP treatment (*n* = 5) (**H**). MDA and Fe^2+^ levels in tumor tissues were detected. After DDP treatment, both MDA and iron content in the tissues increased, and silencing SLC25A10 further increased their levels (**I**, **J**). Electron microscopy of mouse tumor tissue revealed that mitochondria exhibited characteristics of ferroptosis after DDP treatment or SLC25A10 silencing. The SLC25A10 knockdown group receiving DDP treatment showed the most significant mitochondrial shrinkage (**K**). Representative immunohistochemical staining for SLC25A10, Ki67, and 4-HNE in xenograft tumor tissues revealed that the lipid peroxidation product 4-HNE was increased in DDP-treated SLC25A10-knockdown tumors and that the expression of the associated tumor cell proliferation antigen Ki67 was significantly decreased (**L**). The mRNA and protein levels of GPX4, ACSL4, and SLC7A11 were detected in four groups of tumor tissues, and the results were quantified (**M**–**O**). Pattern of induced ferroptosis in CC: When SLC25A10 is downregulated, GSH transport and GPX4 expression are restricted, its protective effect on cells is weakened, intracellular lipid peroxidation occurs, reactive oxygen species accumulate, and ferroptosis is promoted (**P**). Compared with the control group, ns: not significant, *P < 0.05, **P < 0.01, ***P < 0.001, ****P < 0.0001. Scale =100 µm. The data represent the means ± SDs.

We next tested the effect of SLC25A10 on cisplatin sensitivity in vivo using a subcutaneous tumor-bearing mouse model. We injected 1×10^7^ stably transduced (SH-NC and SH1-SLC25A10) HeLa cells subcutaneously into the left axilla of female nude mice. The tumor-bearing mice were then randomly divided into the SH-NC + NS group (*n* = 5), SH-NC + DDP group (*n* = 5), SH1-SLC25A10 + NS group (*n* = 5) and the SH1-SLC25A10 + DDP group (*n* = 5). After 38 days of treatment, we observed that the antitumor effect in the SLC25A10 knockout group (c) was greater than that in the control group (a). The weight and volume of tumors (bd) in the mice treated with DDP were lower than those in the control group (ac), which indicates that DDP exerts antitumor effects on the mice and has toxic side effects. Among these groups, the SLC25A10-knockdown group (d) treated with cisplatin presented the most significant reduction in tumor growth (Fig. 7A–D). The mRNA and protein levels of SLC25A10 in the SLC25A10-knockdown group (cd) were significantly decreased compared to the control group (ab), indicating that SLC25A10 has been effectively knocked out in vivo (Figs. 7E–G and S4).

In addition, the GSH content in the tumors after SLC25A10 knockdown was lower than that in the control group, and the GSH content was further reduced after DDP treatment (Fig. 7H). At the same time, the MDA and Fe^2+^ content in tumors increased due to SLC25A10 silencing, and both levels further increased after DDP treatment (Fig. 7I, J). Under electron microscopy, varying degrees of mitochondrial shrinkage were observed in the SLC25A10 knockdown group and the DDP-treated group, with the most significant changes in mitochondrial ferroptosis observed in the SLC25A10 knockdown group receiving DDP treatment (Fig. 7K). IHC experiments also confirmed that 4-hydroxynonenal (4-HNE), a highly active cytotoxic aldehyde released during the oxidation of unsaturated fatty acids [30], increased lipid peroxidation in SLC25A10-knockdown tumors treated with DDP; in contrast, the tumor cell proliferation antigen Ki67, which reflects cell proliferation, was significantly decreased (Fig. 7L). DDP treatment resulted in a decrease in GPX4 levels in tumor tissue, with a more significant reduction observed after silencing SLC25A10. However, the expression levels of ACSL4 and SLC7A11 were not significantly affected (Fig. 7M–O). This finding was consistent with the results of the in vitro cell experiments, which further confirmed that the downregulation of SLC25A10 restricted GSH transport and GPX4 expression, weakened its protective effect on cells, induced intracellular lipid peroxidation and the accumulation of reactive oxygen species, and promoted ferroptosis (Fig. 7P).

## Discussion

Changes in energy metabolism and oxidation‒reduction homeostasis are often found in tumor cells [31]. Previous studies have shown that SLC25A10 is the main glutathione transporter in mitochondria and that SLC25A10-mediated glutathione transport is essential for mitochondria to maintain ROS homeostasis and normal respiratory function [26, 27]. Research has revealed that SLC25A10 is upregulated in a variety of tumors, which suggests that SLC25A10 is also closely related to tumor development. Consistent with these studies, our data suggest that SLC25A10 expression is elevated in CC and is correlated with the degree of tumor differentiation, lymph node metastasis, and clinical stage in patients. We also found that SLC25A10 silencing inhibited cell proliferation in vitro and xenograft growth in tumor-bearing mice, which suggests that SLC25A10 silencing exerts an antitumor effect on CC. Moreover, the upregulation of SLC25A10 induced cisplatin resistance in cervical cancer cells.

Programmed cell death (PCD), which is an inborn process that ensures cell homeostasis, can be activated by cytokines, radiation, and oxidative stress, among other factors. In cancer cells, intrinsic resistance to PCD (such as apoptosis and ferroptosis) is necessary to ensure high viability and is responsible for resistance to cytotoxic chemotherapy [32]. Ferroptosis has been described as a type of oxidative PCD that is dependent on iron and ROS, the dysregulation of which allows cancer cells to survive [33]. The ability of cancer cells to initiate apoptosis and ferroptosis depends on the regulation of the expression of oncogenes that inhibit cancer cell death [34]. The induction of ferroptosis by knockdown of oncogenes is a promising new method for eradicating malignant cells [35, 36]. Recent studies have shown that inhibiting ferroptosis can promote resistance to 5-FU in colorectal cancer cells [37, 38]. In this study, RNA-seq suggested that SLC25A10 is associated with ferroptosis, lipid metabolism, and oxidation‒reduction reactions (Fig. 4A, B). SLC25A10 was able to reduce cell death under DDP exposure, but this phenomenon could be reversed by ferroptosis inhibitors, which suggests that ferroptosis may occur in cervical cancer cells induced by DDP and that SLC25A10 has an inhibitory effect on ferroptosis.

We further examined the main indicators of ferroptosis in cells and in vivo xenograft tumors and found that silencing SLC25A10 resulted in glutathione imbalance, increased lipid peroxidation, ROS accumulation, Fe^2+^ aggregation, and mitochondrial morphological changes, all of which indicated the occurrence of ferroptosis. GPX4 is one of the most important antioxidant enzymes and a crucial regulator of cancer cell apoptosis. Activation of GPX4 can inhibit ferroptosis and inflammation [39, 40]. In this study, the expression of GPX4 was affected by DDP, further indicating that DDP can induce ferroptosis in CC. Additionally, GPX4 is positively regulated by SLC25A10.Therefore, we believe that SLC25A10 can inhibit ferroptosis and promote DDP resistance in cervical cancer cells. Additionally, GSH is a main cofactor of GPX4, so GSH regulation can also indirectly modulate GPX4 [41]. As mentioned above, SLC25A10 is the main glutathione transporter in mitochondria. We speculate that the mechanism by which upregulated SLC25A10 inhibits ferroptosis involves promoting the transport of GSH and the expression of GPX4, which enhances its protective effect on cells, reduces intracellular lipid peroxidation and the accumulation of reactive oxygen species, inhibits ferroptosis, and thus enhances resistance to DDP in cervical cancer cells. Our findings suggest that targeting SLC25A10 combined with DDP may be an effective tumor therapy.

## Conclusion

SLC25A10 can serve as a prognostic marker of CC and an effective therapeutic target for the treatment of platinum-based drug resistance. For this reason, more preclinical and clinical studies are necessary to improve the treatment of cisplatin-resistant CC.

## Materials and methods

### Data source

The gene expression data of cervical cancer patients were obtained from the TCGA dataset and the GEO dataset (GSE9750). For further details regarding the GEO dataset, please visit https://www.ncbi.nlm.nih.gov/geo/query/acc.cgi?acc=GSE9750. During the analysis, batch effects were initially removed. Differential expression analysis was then performed on 33 tumor tissue samples and 24 normal cervical epithelial tissue samples using the limma package in R. Ultimately, the potential role of the SLC25 family in tumorigenesis was assessed using the GSEA algorithm.

### Human tissue

In all, 12 pairs of cervical cancer tissues and matched normal cervical epithelial tissues from patients undergoing surgical treatment at The Affiliated Zhangjiagang Hospital of Soochow University and 55 cases of cervical tissue in paraffin blocks from the pathology department archives were examined. This study was approved by the Medical Ethics Committee of The Affiliated Zhangjiagang Hospital of Soochow University. Each patient provided written informed consent. Patient information is provided in Table 2.

**Table 2:** Information on the cervical cancer patients.

No.	Sex	Age	Histopathological type	Differentiation	Lymph node metastasis	FIGO Stage	IHC immunoscore
1	Female	51	Adenocarcinoma	Low	Yes	IIIC1	6
2	Female	50	Squamous	Mid	No	IIA	4
3	Female	62	Squamous	Mid	Yes	IIIC1	4
4	Female	52	Squamous	Mid	No	IB1	3
5	Female	46	Squamous	Mid	No	IB2	3
6	Female	51	Squamous	Mid	No	IA2	2
7	Female	51	Squamous	Mid	No	IIA2	4
8	Female	48	Squamous	Mid	No	IIA1	4
9	Female	44	Squamous	Mid to Low	No	IIA2	4
10	Female	54	Squamous	High	No	IB1	2
11	Female	58	Squamous	High	No	IB1	2
12	Female	68	Squamous	Mid	No	IA2	2
13	Female	51	Squamous	Mid to Low	No	IB1	2
14	Female	59	Squamous	Mid	No	IIA1	4
15	Female	51	Squamous	High	No	IB1	2
16	Female	61	Squamous	Mid to Low	Yes	IIA2	4
17	Female	52	Adenocarcinoma	Mid	No	IB1	2
18	Female	54	Squamous	Mid to Low	No	IB1	3
19	Female	72	Squamous	Mid to Low	Yes	IIA1	4
20	Female	48	Adenocarcinoma	Mid	No	IA2	2
21	Female	70	Squamous	Mid	No	IB1	2
22	Female	73	Adenocarcinoma	Mid to Low	No	II	4
23	Female	61	Squamous	Mid to Low	No	IB2	3
24	Female	64	Squamous	Mid	No	IIA	3
25	Female	54	Adenosquam	Mid to Low	Yes	IIA1	4
26	Female	56	Squamous	Mid	Yes	IIIB	6
27	Female	36	Squamous	Mid	No	IIA1	3
28	Female	58	Squamous	Mid to Low	Yes	IB2	3
29	Female	61	Squamous	Mid	No	IIA2	4
30	Female	51	Squamous	Mid	No	IB1	2
31	Female	40	Squamous	Mid	No	IB3	2
32	Female	80	Adenocarcinoma	Mid	No	IIA2	4
33	Female	45	Adenocarcinoma	Mid	No	IIA1	4
34	Female	58	Squamous	Mid	No	IB3	3
35	Female	53	Squamous	Mid	No	IIA2	4
36	Female	83	Squamous	Low	No	IB2	4
37	Female	59	Squamous	Mid	No	IIA1	4
38	Female	68	Adenocarcinoma	Mid	No	IB2	2
39	Female	53	Adenocarcinoma	Mid	No	IB1	2
40	Female	57	Adenocarcinoma	Mid	Yes	IVB	6
41	Female	59	Squamous	Mid	No	IB3	2
42	Female	33	Squamous	Mid	No	IB3	2
43	Female	78	Squamous	Mid	Yes	IIB	4
44	Female	77	Squamous	Mid	No	IB3	3
45	Female	75	Adenocarcinoma	Mid	No	IB1	2
46	Female	45	Squamous	Mid to Low	Yes	IIIC1	4
47	Female	51	Squamous	Mid	No	IIA2	2
48	Female	71	Squamous	Mid	No	IIA	2
49	Female	72	Adenocarcinoma	Low	Yes	IIIC1	9
50	Female	69	Squamous	Mid	No	IB2	3
51	Female	47	Squamous	Mid to Low	Yes	IIA2	4
52	Female	48	Squamous	Low	Yes	IIIC2	4
53	Female	53	Adenocarcinoma	Mid	Yes	IB2	2
54	Female	62	Squamous	Mid to Low	Yes	IIA2	4
55	Female	49	Adenocarcinoma	Low	No	IB2	4
56	Female	59	Adenocarcinoma	Mid to Low	No	IIA2	4
57	Female	71	Squamous	Low	No	IIA1	4
58	Female	54	Adenocarcinoma	Low	No	IB2	3
59	Female	39	Squamous	Mid	Yes	IIA1	4
60	Female	53	Squamous	Mid	No	IB2	2
61	Female	50	Undifferentiated	Low	No	IB1	3
62	Female	37	Adenocarcinoma	Mid	Yes	IB3	9
63	Female	56	Adenosquam	Mid to Low	Yes	IIA2	6
64	Female	76	Squamous	Low	No	IB	4
65	Female	73	Squamous	Mid	No	IIA1	3
66	Female	67	Squamous	Mid	No	IIA2	4
67	Female	72	Squamous	Low	Yes	IIA	4

### Cell culture, transfection and reagents

CaSki and HeLa (human cervical cancer cell lines) cells obtained from the Cell Bank of Institute of Biological Sciences of CAS (Shanghai, China) were cultured in RPMI 1640 medium (1640, Gibco, USA) or Dulbecco’s Modified Eagle’s Medium (DMEM, Gibco, USA) supplemented with 10% fetal bovine serum (FBS, Gibco, USA) and antibiotics (100 mg/mL penicillin‒streptomycin, Beyotime, China) at 37 °C in a humidified atmosphere (90%) containing 5% CO_2_. Lentiviral particles carrying SH1-SLC25A10, SH2-SLC25A10, and OV-SLC25A10 and the corresponding empty vectors SH-NC and OV-NC were constructed by Gekkai Gene Co., Ltd. Transfections were performed with a lentivirus transduction system according to the manufacturer’s instructions. RSL3, ferrostatin 1 (Ferr-1), necrosulfonamide (Nec), and Z-VAD-FMK (Z-V) were purchased from MCE. Erastin and cisplatin (DDP) were purchased from Beyotime.

### Immunohistochemistry

Tissue sections were subjected to immunohistochemistry (IHC) according to a previously published protocol [42], after which quantitative analysis was performed with ImageJ software [43, 44]. The positive cells and the total number of cells were counted in 5 randomly selected fields (×200) under a microscope, and brown‒yellow particles were observed in the SLC25A10-positive cells. The percentages of positive cells among the total number of cells were ≤10%, 11–25%, 26–50%, and >50%, which corresponded to scores of 0, 1, 2, and 3 points, respectively. The staining intensity of the cells was scored as follows: no staining, 0 points; light yellow, 1 point; light brown, 2 points; and brown, 3 points. The IHC immunoscore was calculated according to the following formula: IHC immunoscore = the proportion of positive cells × the staining intensity. A score of 0–2 points was considered negative, and a score of 3–9 points was considered positive [45]. SLC25A10, α-tubulin, 4-hydroxynonenal (4-HNE), and Ki67 antibodies were purchased from Bioss. GPX4, ACSL4 antibodies were purchased from Proteintech. The SLC7A11 antibody used for the OV-NC and OV-SLC25A10 groups was sourced from Abcam (ab307601, UK). All other SLC7A11 antibodies used in this manuscript were obtained from Proteintech (26864-1-AP, China).

### Glutathione (GSH) and malondialdehyde (MDA) assays

GSH and GSSG assay kits (Beyotime, China) were used to determine the total glutathione level in vivo and in vitro [46]. The MDA content was measured via a malondialdehyde (MDA) content assay kit (Beyotime, China) according to the manufacturer’s instructions [47]. The protein concentration was determined with a BCA protein assay kit (Thermo Fisher, USA), and the total glutathione and MDA contents per milligram of protein or tissue were calculated.

### Ferrous ion detection

After different treatments, cells were rinsed three times with PBS and then treated with ferrous ion detection kit (Elabscience, China). After incubated at 37 °C incubator for 1 h, the cells were washed three times with PBS. The results were observed by fluorescence microscope.

### Measurement of the Fe^2+^ levels

The cortex was prepared into 10% tissue homogenate and centrifuged, and the supernatant was used to follow-up experiments. The Fe^2+^ level was measured using ferrous iron colorimetric assay kit (E-BC-K773-M, Elabscience, China) according to the manufacturer’s instructions.

### Oxygen species (ROS) assay

Changes in intracellular ROS levels were measured using an ROS detection kit (Beyotime, China). The cells were seeded in a 6 cm petri dish (1×10^6^ cells/well) and were allowed to adhere overnight. The cells were incubated with 10 µM erastin or control medium for 24 h, after which they were washed 3 times in PBS and incubated with DCFH-DA at 37 °C for 20 min. The ROS-positive cells appeared red under a fluorescence microscope [42].

### TEM

The ultrastructure of the mitochondria was analyzed by transmission electron microscopy. SLC25A10-overexpressing or control HeLa cells were treated with DMSO or 10 μM erastin, fixed in 2.5% glutaraldehyde at 4 °C for 2.5 hours, washed 3 times in PBS, and postfixed in 1% OsO4 at 4 °C for 2 hours. The sample was then dehydrated through an ethanol gradient and subsequently embedded in Spurr’s resin. Ultrathin sections were then collected and stained with either uranyl acetate or lead citrate and examined by transmission electron microscopy [48].

### Animal studies

Female nude mice (18 ± 2 g, 4 weeks old) were provided by Hangzhou Ziyuan Laboratory Animal Technology Co., Ltd (SCXK2024-0004). The nude mice were maintained under specific pathogen-free (SPF) conditions for one week, and then, 1 × 10^7^ stably transduced (SH-NC and SH1-SLC25A10) HeLa cells were injected subcutaneously into the left axilla. Eight days after the tumor-bearing model was established (day 0), DDP was dissolved in 0.9% normal saline (NS) and administered by intraperitoneal injection at a dose of 3 mg/kg mouse body weight every 3 days for 38 consecutive days. The tumor volume (V) was measured every 3 days and was calculated according to the following formula: *V* = 0.52 × length × width × width. After the mice were sacrificed, the tumors were removed and weighed for further study.

### Other experiments

Other assays, including CCK-8 (detection of cell viability), scratch and colony formation assays, have been previously reported [49, 50]. The protocols for real-time quantitative RT‒PCR (qRT‒PCR) and Western blotting for the detection of mRNAs and proteins, respectively, have also been previously reported [42]. The primers were designed by Sangon Biotech and are listed in Table S1. The uncropped, imprinted images are provided in the Supplementary Material (Western Blots).

### Statistical analysis

All experiments were repeated at least 3 times. GraphPad Prism 9 was used for statistical analysis. Data were normally distributed and were presented as mean ± standard deviation (SD). For statistical analysis, unpaired t-tests (two-tailed) were employed for single comparisons, while two-way ANOVA was utilized for multiple comparisons. Additionally, the χ² test was conducted to compare categorical data between groups.

## Supplementary information

Supplemental Material: Table S1 and Figure S1 to S4.

Figure S1

Figure S2

Figure S3

Figure S4

Supplemental Material Western Blots: The uncropped blotting images of the study.

## Data Availability

All data needed to evaluate the conclusions in the paper are present in the paper and the Supplementary Materials. The RNA-seq date have been deposited into CNGB Sequence Archive(CNSA) of China National GeneBank DataBase with accession number CNP0007158.
