# Supplementary material for: SLC25A10 promotes cisplatin resistance by inhibiting ferroptosis in cervical cancer
Source: Cell Death Discov. 2025 Oct 7;11:447. doi: 10.1038/s41420-025-02712-5 (PMC12504685; doi:10.1038/s41420-025-02712-5)
Supplement: Supplementary file 1 — Supplemental Material: Table S1 and Figure S1 to S4. [file 41420_2025_2712_MOESM1_ESM.docx]

Supplementary Materials for

**SLC25A10 promotes cisplatin resistance by inhibiting ferroptosis in cervical cancer**

Chenglei Ma *et al.*

Corresponding author. Email: huanghaiwei760401@163.com (Prof. Haiwei Huang)

**This PDF file includes:**

Table S1

Figure S1 to S4

**Tables and Figures**

Table S1 The sequences of primers used were as follows

| Primer | Sequences(5’ to 3’) |
| --- | --- |
| Human-GAPDH-F | CAGGAGGCATTGCTGATGAT |
| Human-GAPDH-R | GAAGGCTGGGGCTCATTT |
| SLC25A5-F | TTATAGACTGCGTGGTCCGTA |
| SLC25A5-R | GGCGAAGTTAAGAGCCTGGG |
| SLC25A8-F | ACTGTGCCCTTACCATGCTC |
| SLC25A8-R | AGGAGAGGCTCAGAAGGGAG |
| SLC25A10-F | CTACTCCCTGACTCGGTTCG |
| SLC25A10-R | TCCTGACGTTGACCAAGTCTG |
| SLC25A39-F | AGAGCTGGCTCAATGGGTTC |
| SLC25A39-R | GACCTGGCGTTGGGTCTTTA |
| SLC25A50-F | GCTGTGTAACTCACTGGCCT |
| SLC25A50-R | ACCACAGTTGTTGACAGCCA |
| Human-ACSL4-F | ACCAGGGAAATCCTAAGTGAAG |
| Human-ACSL4-R | GGTGTTCTTTGGTTTTAGTCCC |
| Human-GPX4-F | ATGGTTAACCTGGACAAGTACC |
| Human-GPX4-R | GACGAGCTGAGTGTAGTTTACT |
| Human-SLC7A11-F | TTACCAGCTTTTGTACGAGTCT |
| Human-SLC7A11-R | GTGAGCTTGCAAAAGGTTAAGA |

**Figure S1.** We conducted Gene Set Enrichment Analysis (GSEA) using the 53 genes of the SLC25 family as a gene set. The GSEA results indicated that the SLC25 family is significantly associated with cervical cancer (P = 0.02995) and may exert cancer promotion. The gene expression data of cervical cancer patients were obtained from the GEO dataset (GSE9750) (<https://www.ncbi.nlm.nih.gov/geo/query/acc.cgi>?acc=GSE9750) (A).Differentially expressed members of mitochondrial SLC25 between cervical cancer and normal tissue in TCGA-CESC(http://gepia.cancer-pku.cn).* |Log2FC|<1, p-value<0.01 (B).

**Figure S2.** Detection of mRNA expression of 5 differentially expressed members of mitochondrial SLC25 in cervical cancer tissues (Tumor) and matched normal cervical epithelial tissues (Normal) of 12 primary cervical cancer patients (n=12). * Compared with "T", P<0.05.

**Figure S3.** Investigate the effect of cisplatin on the expression of GPX4, ACSL4, and SLC7A11 in HeLa cell lines by detecting mRNA levels, and quantify the results.

**Figure S4.** The Western Blot bands of tissues from two nude mice have been presented in the text. The bands shown here are the supplementary data for the remaining mouse tissues, with the corresponding quantitative analysis in Figure 7G.
