# Supplementary figures and images for: SLC25A10 promotes cisplatin resistance by inhibiting ferroptosis in cervical cancer
Source: Cell Death Discov. 2025 Oct 7;11:447. doi: 10.1038/s41420-025-02712-5 (PMC12504685; doi:10.1038/s41420-025-02712-5)

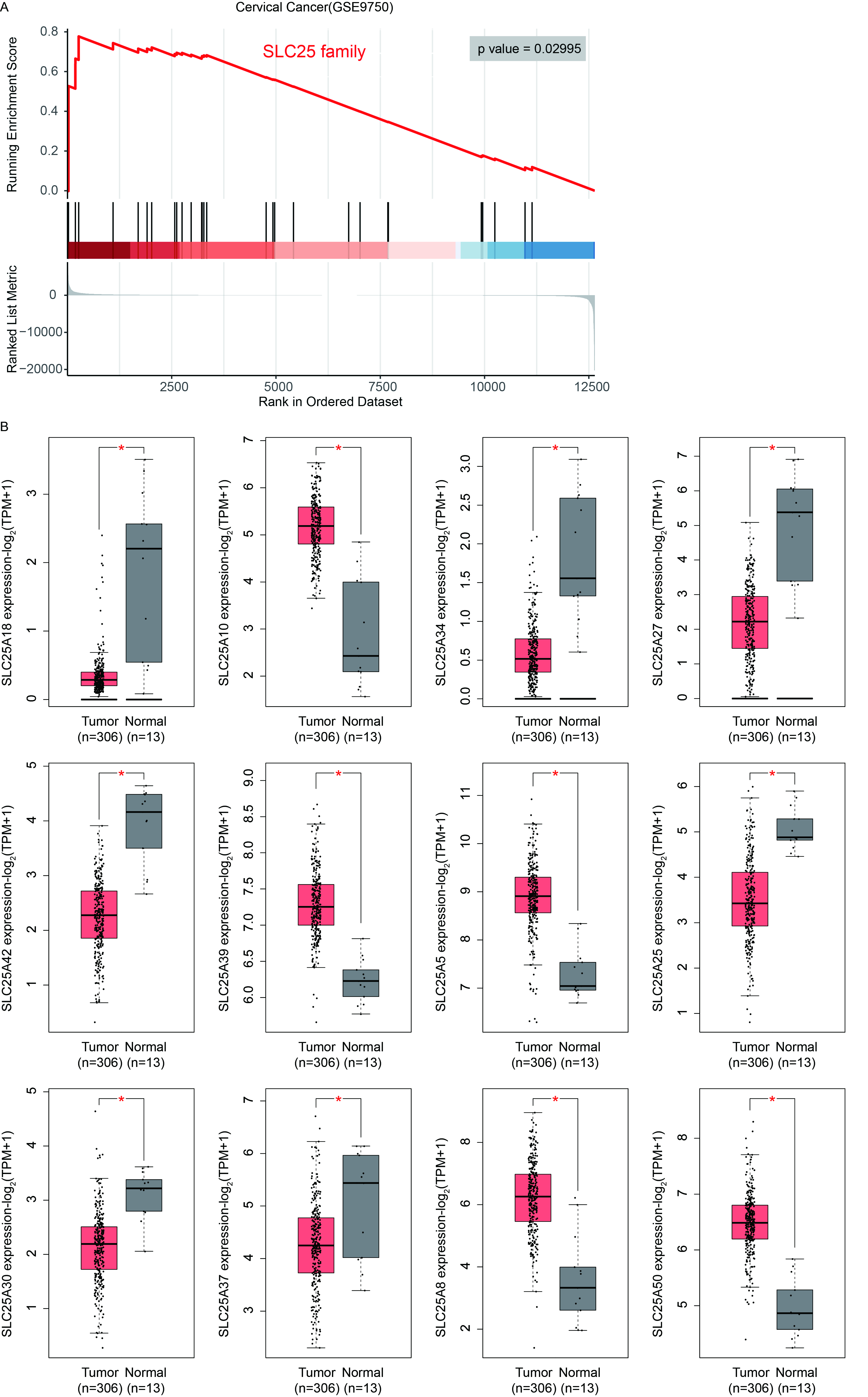

Supplement: Supplementary file 2 — Figure S1 [file 41420_2025_2712_MOESM2_ESM.tif]

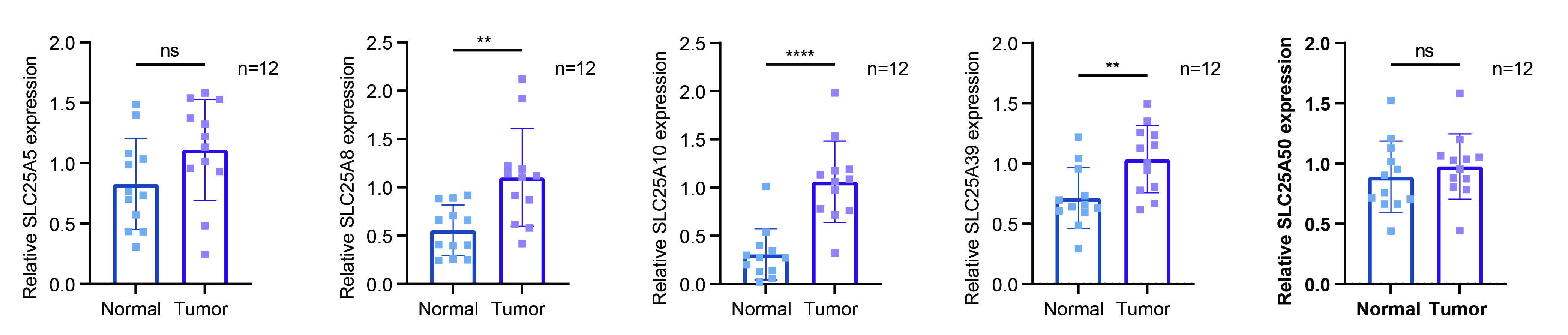

Supplement: Supplementary file 3 — Figure S2 [file 41420_2025_2712_MOESM3_ESM.tif]

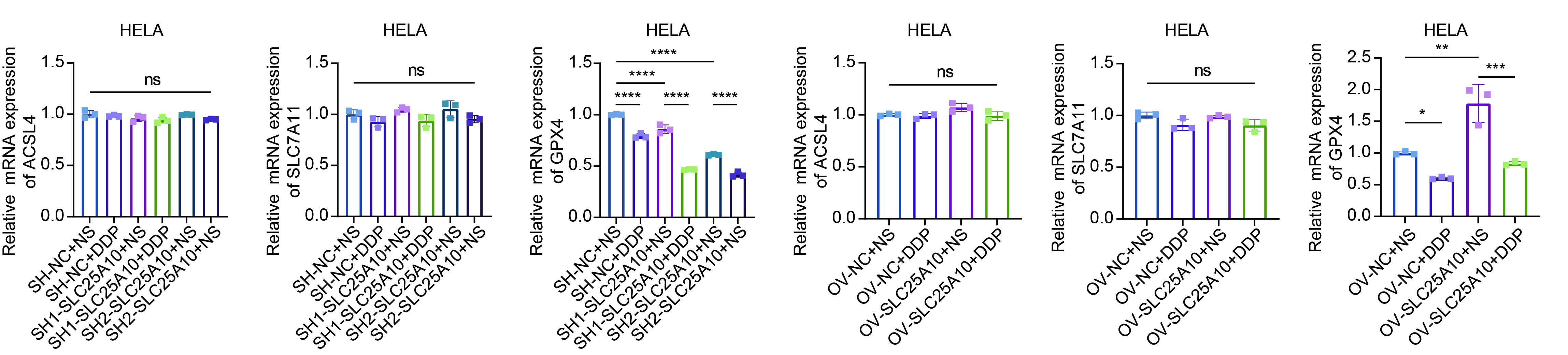

Supplement: Supplementary file 4 — Figure S3 [file 41420_2025_2712_MOESM4_ESM.tif]

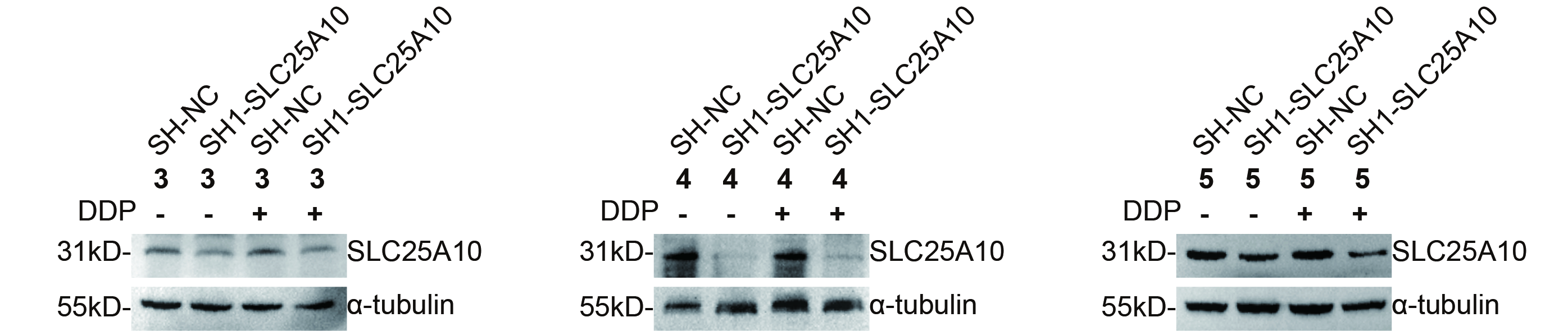

Supplement: Supplementary file 5 — Figure S4 [file 41420_2025_2712_MOESM5_ESM.tif]

The uncropped blotting images of the study.

Figure 1C

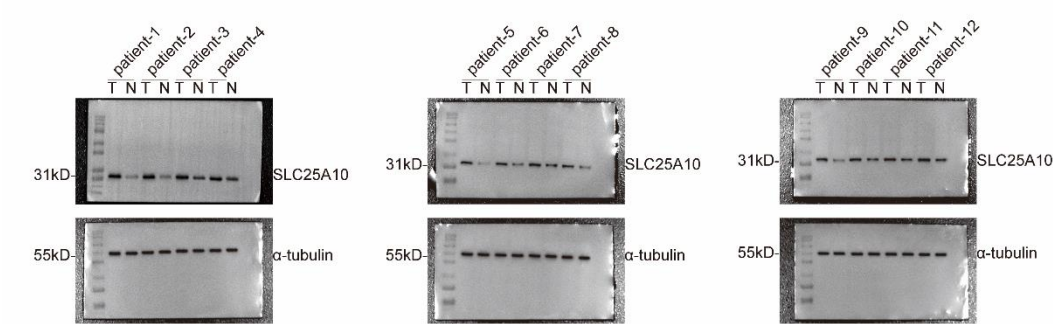

Figure 2B

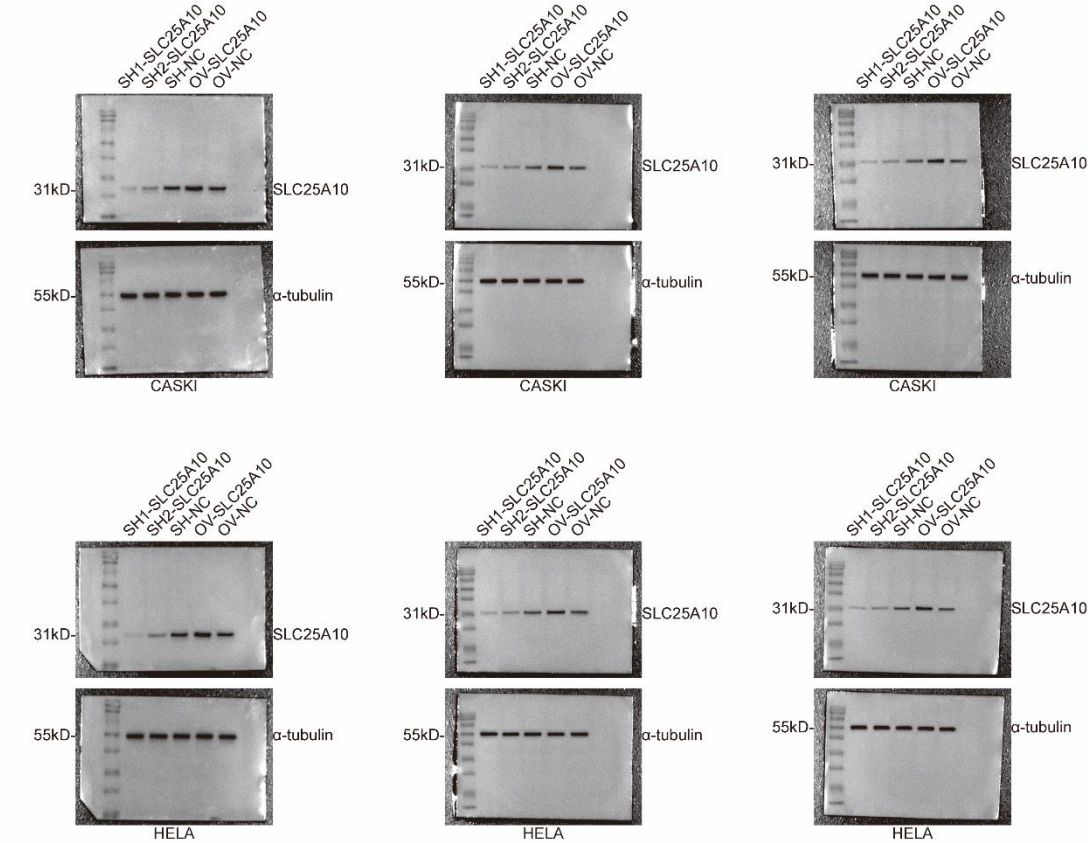

Figure 6L

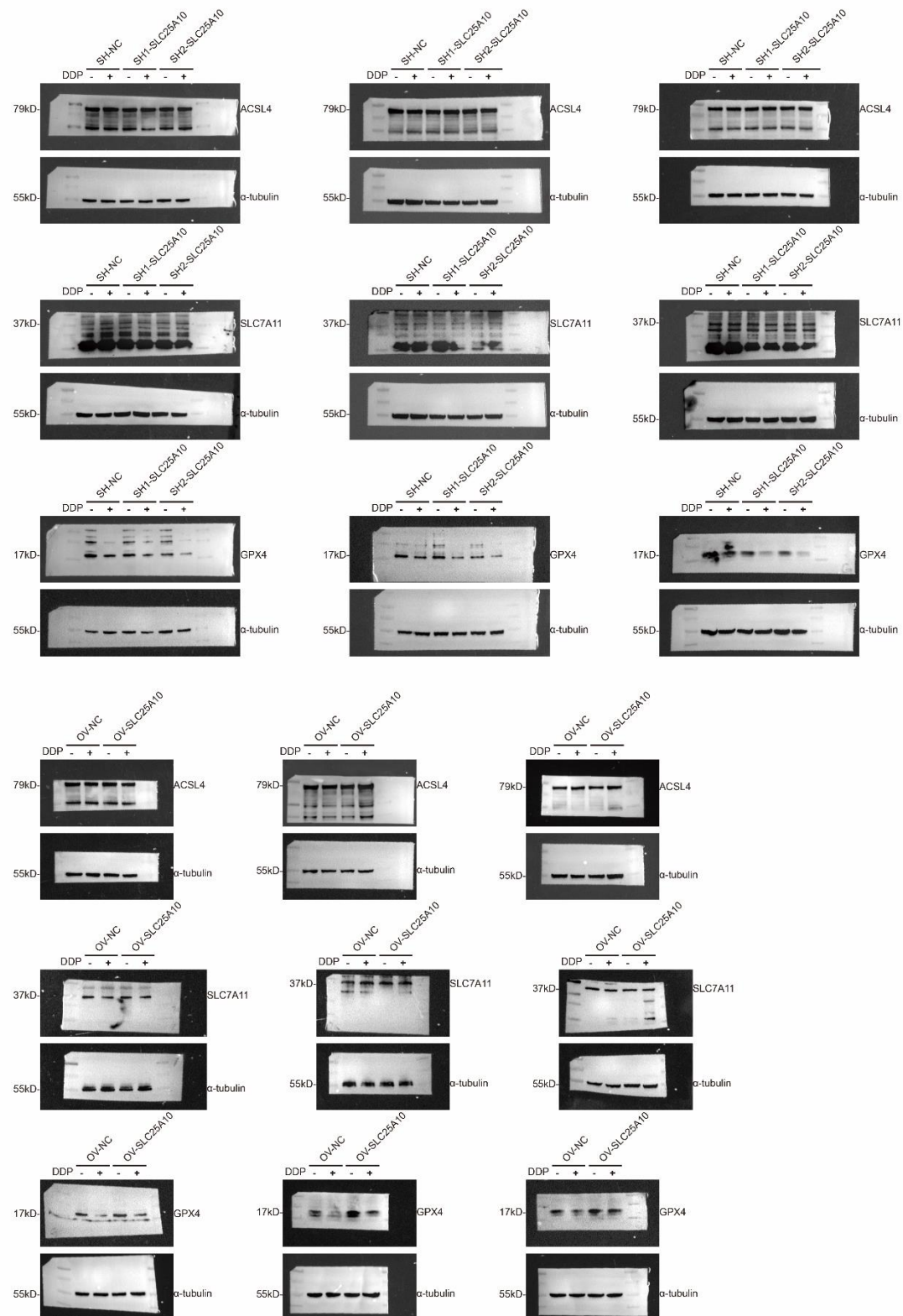

Figure 7F

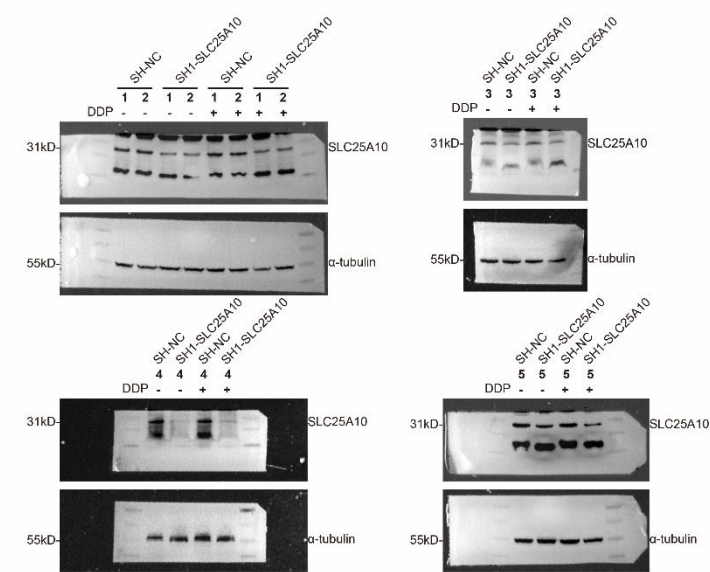

Figure 7N

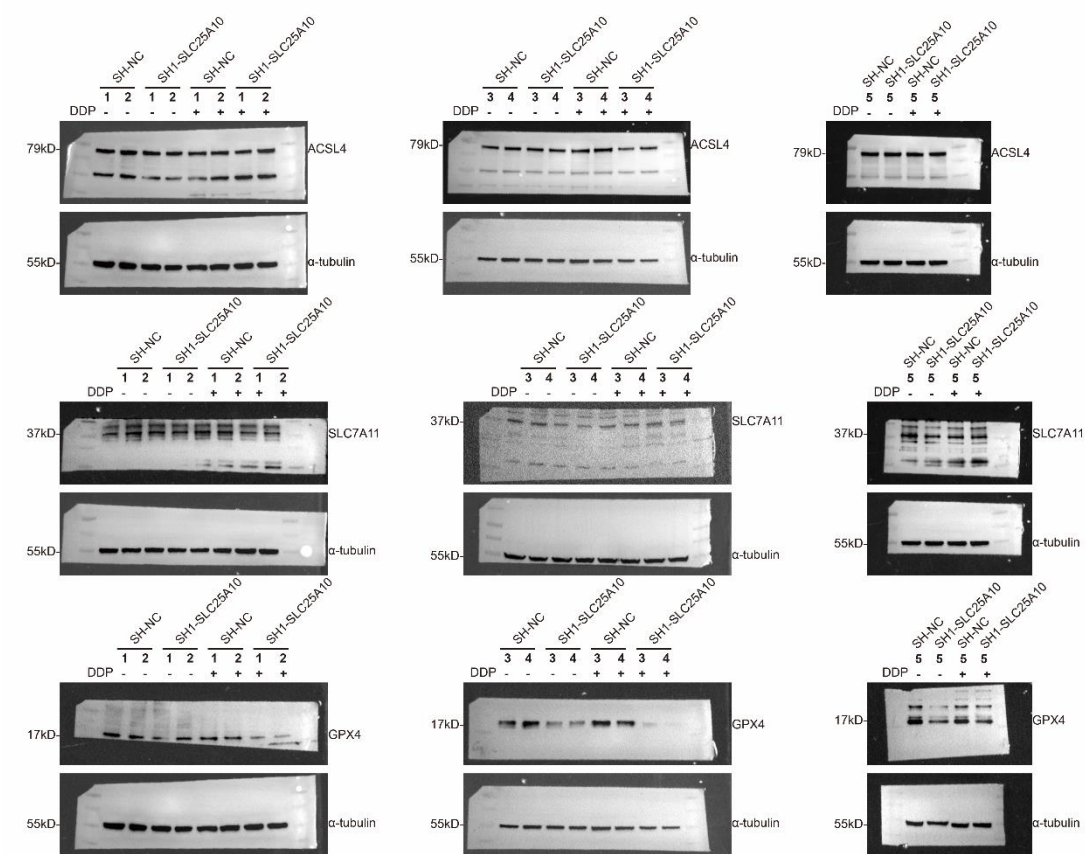

Supplement: Supplementary file 6 — Supplemental Material Western Blots: The uncropped blotting images of the study. [file 41420_2025_2712_MOESM6_ESM.pdf]
